# Validation and delineation of a locus conferring *Fusarium* crown rot resistance on 1HL in barley by analysing transcriptomes from multiple pairs of near isogenic lines

**DOI:** 10.1186/s12864-019-6011-8

**Published:** 2019-08-14

**Authors:** Shang Gao, Zhi Zheng, Jonathan Powell, Ahsan Habib, Jiri Stiller, Meixue Zhou, Chunji Liu

**Affiliations:** 1 CSIRO Agriculture and Food, St Lucia, Queensland 4067 Australia; 2 School of Land and Food and Tasmanian Institute of Agriculture, University of Tasmania, Hobart, Australia; 3 Biotechnology and Genetic Engineering Discipline, Khulna University, Khulna, 9208 Bangladesh

**Keywords:** *Fusarium* crown rot, QTL validation, Near-isogenic line, RNA-seq, Transcriptome, Barley

## Abstract

**Background:**

*Fusarium* crown rot (FCR) is a chronic and severe disease in cereal production in semi-arid regions worldwide. A putative quantitative trait locus conferring FCR resistance, *Qcrs.cpi-1H,* had previously been mapped on the long arm of chromosome 1H in barley.

**Results:**

In this study, five pairs of near-isogenic lines (NILs) targeting the 1HL locus were developed. Analysing the NILs found that the resistant allele at *Qcrs.cpi-1H* significantly reduced FCR severity. Transcriptomic analysis was then conducted against three of the NIL pairs, which placed the *Qcrs.cpi-1H* locus in an interval spanning about 11 Mbp. A total of 56 expressed genes bearing single nucleotide polymorphisms (SNPs) were detected in this interval. Five of them contain non-synonymous SNPs*.* These results would facilitate detailed mapping as well as cloning gene(s) underlying the resistance locus.

**Conclusion:**

NILs developed in this study and the transcriptomic sequences obtained from them did not only allow the validation of the resistance locus *Qcrs.cpi-1H* and the identification of candidate genes underlying its resistance, they also allowed the delineation of the resistance locus and the development of SNPs markers which formed a solid base for detailed mapping as well as cloning gene(s) underlying the locus.

**Electronic supplementary material:**

The online version of this article (10.1186/s12864-019-6011-8) contains supplementary material, which is available to authorized users.

## Background

*Fusarium* crown rot (FCR), caused mainly by *F. pseudograminearum*, is a severe and chronic disease of cereals in semi-arid cropping regions worldwide [1, 2]. To reduce FCR damage, several agronomic measures have been developed. They include crop rotation and stubble management [3, 4]. These practices can reduce the impact of FCR in certain circumstances but are not always useful due to economic and practical requirements [5]. It has long been recognised that growing resistant varieties is an essential component to effectively manage this disease [6].

The approach of identifying and transferring major QTL into elite genotype has been used in breeding FCR-resistant varieties in wheat and barley [7, 8]. Up to date, four putative QTL conferring FCR resistance have been reported in barley [9]. They locate on chromosome arms 1HL [10], 3HL [11], 4HL [12] and 6HL [13], respectively. Similar to those noticed in wheat [14, 15], strong interactions between FCR severity and other characteristics including flowering time [12, 16] and plant height [11, 17] have also been detected in barley. The FCR resistance locus on chromosome arm 3HL in barley also co-locates with gene(s) controlling spike structure [18]. Results from previous studies also showed that water availability affects FCR development [19].

The interactions between FCR severity and other characteristics indicate that QTL detected through mapping can only be treated as putative. The effectiveness of a QTL detected from segregating populations needs to be validated. Near isogenic lines (NILs) have been used widely in validating QTL for various characteristics [20, 21]. They were also used to validate QTL conferring resistance to FCR in cereals [22, 23].

The main focus of transcriptomic analysis was to detect differentially expressed genes (DEGs) when the technique was initially introduced [24, 25]. The analysis is now also widely used to uncover genetic markers for various purposes [26, 27]. Combined with the use of NILs, distributions of variations detected from transcriptomic sequences have been exploited effectively in validating QTL and obtaining markers for fine mapping targeted loci [28–30].

In the study reported here, NILs were developed and used to validate the QTL conferring FCR resistance on 1HL. Transcriptomic sequences were then obtained from three pairs of the NILs. Shared SNPs detected from the transcriptomic sequences among the NIL pairs were used to further delineate the QTL interval and identify candidate genes underlying the resistance locus on 1HL.

## Results

### Development and validation of NILs targeting the FCR resistance locus on 1HL

Eight heterozygous plants were initially selected from the two segregating populations based on the profiles of the SSR marker *WMC1E8*. A single pair of putative NILs was obtained from each of the heterozygous plants. Significant difference in morphology between any pairs of the putative ‘R’ and ‘S’ isolines was not observed. Significant difference in FCR severity was detected between the isolines for five of the eight putative NIL pairs. As expected, the isolines carrying the resistant allele from the donor parent AWC079 always gave much lower FCR severity than their counterparts (Table 1). The average DI for the ‘R’ isolines was 27.1, whereas it was 68.4 for the ‘S’ isolines. Three of the five NIL pairs with the largest difference in FCR severity, namely 1H_NILs: 1H_NIL1, 1H_NIL2 and 1H_NIL3, were selected and used for RNA-seq analysis.

**Table 1:** Difference in disease index between the resistant and susceptible isolines for the five NIL pairs targeting the 1HL locus conferring FCR resistance

NIL^a^	Genetic Background	DI Mean^b^	SE^c^	Difference (%)^d^	*P* value^e^
1H_NIL1_R	Lockyer//AWCS079/AWCS276 F8	24.9	4.2	66.1	< 0.01
1H_NIL1_S		73.7	6.4		
1H_NIL2_R	Lockyer//AWCS079/AWCS276 F8	24.6	2.1	63.4	< 0.01
1H_NIL2_S		67.3	4.1		
1H_NIL3_R	Commander//AWCS079/AWCS276 F8	26.4	1.8	58.0	< 0.01
1H_NIL3_S		62.9	2.6		
1H_NIL4_R	Lockyer//AWCS079/AWCS276 F8	27.9	1.0	57.4	< 0.01
1H_NIL4_S		65.5	1.4		
1H_NIL5_R	Commander//AWCS079/AWCS276 F8	31.7	2.5	56.4	< 0.01
1H_NIL5_S		72.7	4.8		

^a^ ‘R’ represents isolines with the allele from the resistant parent ‘AWC079’ and ‘S’ isolines with an alternative allele from the susceptible parents

^b^ The mean of disease index (DI value) observed from four trials for each isoline

^c^ ‘*SE*’ represents standard error

^d^ Differences between DI values of ‘R’ and ‘S’ isolines

^e^ *‘P* value’ was generated with the student’s *t* test

### Transcriptome analyses

A total of 792 million quality reads were generated from the 36 samples (see the section of Materials and methods) with an average of 22 million reads per sample. The reads from each of the samples covered on average 21,571 high confidence (HC) genes (54.2% of all HC genes) based on the genome of Morex.

**Fig. 1:**
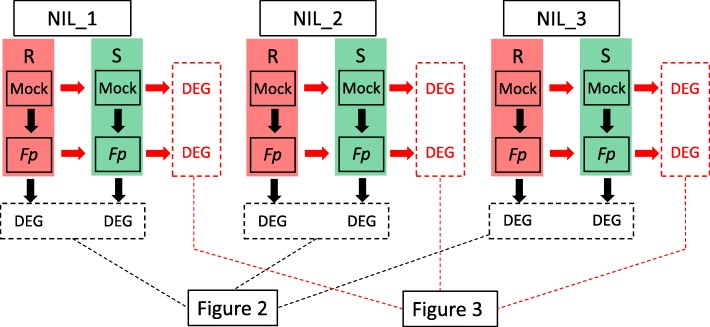
The experimental design for differential gene expression analysis

**Fig. 2:**
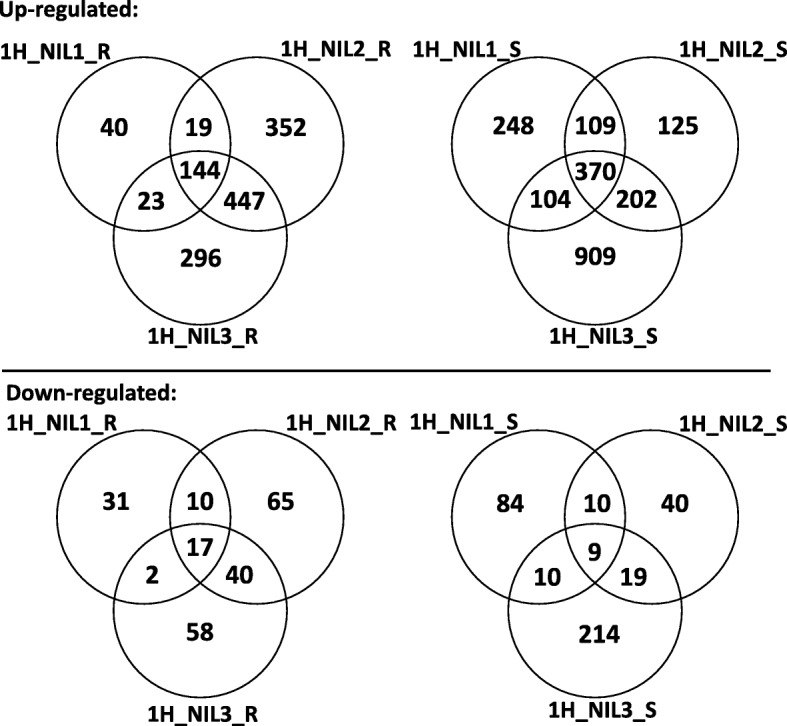
DEGs for each of the 1H_NIL pairs following *Fp*- and mock-inoculation (R^M^_vs_R^I^ and S^M^_vs_S^I^). Venn diagrams in upper panel show the numbers of up-regulated DEGs in each ‘R’ (left) and ‘S’ (right) isolines. Venn diagrams in lower panel show the numbers of down-regulated DEGs in each ‘R’ (left) and ‘S’ (right) isolines. DEGs were determined with the threshold of FDR ≤ 0.05 and |log_2_ fold-change| ≥ 1 or ‘inf’ (one of the comparative objects did not express and the other did)

To analyse host response to *Fusarium* infection, DEGs were detected between *Fp*- (*F. pseudograminearum-*) and mock-inoculated samples of the same isoline. This analysis identified a total of 1323 DEGs from the ‘R’ isolines and 2083 from the ‘S’ isolines. The numbers of up-regulated genes were significantly higher than those down-regulated ones following *Fp*-inoculation (Table 2). Of the up-regulated genes, 144 were shared by all the three ‘R’ isolines and 370 by the three ‘S’ isolines (Figs. 1 and 2). Of the down-regulated genes, 17 were shared by the three ‘R’ lines and only 9 by the three ‘S’ lines. Expression patterns consistent with the RNA-seq analysis were obtained in the qRT-PCR analysis for each of the three genes assessed (Additional file 1: Table S1).

**Table 2:** Number of differentially expressed genes (DEGs) identified from all pairwise comparisons

NIL pair	Comparison^a^	Number of DEGs	
		Up-regulated	Down-regulated
1H_NIL1	R^M^_vs_R^I^	226	60
	S^M^_vs_S^I^	831	113
1H_NIL2	R^M^_vs_R^I^	962	132
	S^M^_vs_S^I^	806	78
1H_NIL3	R^M^_vs_R^I^	910	117
	S^M^_vs_S^I^	1585	252
1H_NIL1	R^I^_vs_S^I^	48	236
	R^M^_vs_S^M^	225	123
1H_NIL2	R^I^_vs_S^I^	51	459
	R^M^_vs_S^M^	178	89
1H_NIL3	R^I^_vs_S^I^	249	132
	R^M^_vs_S^M^	80	71

^a^ ‘*M*’ stands for ‘mock-inoculation’, ‘*I*’ for *Fp*-inoculation, ‘*R*’ for resistant isolines and ‘*S*’ for susceptible isolines

Gene ontology (GO) term enrichment analysis was performed on sets of differentially expressed genes from each comparison, separating out upregulated from downregulated genes. The goal of this approach was to isolate particular biological processes which might explain the difference in resistance levels observed between ‘R’ and ‘S’ isolines. For genes up- or down-regulated during infection in ‘R’ isolines (‘R^M^ vs R^I^’), 11, 17 and 12 enriched terms were identified for 1H_NIL1_R, 1H_NIL2_R and 1H_NIL3_R, respectively (Additional file 2: Table S2). When observing genes upregulated during infection in susceptible isolines (‘S^M^ vs S^I^’), a total of six, nine and fifteen enriched terms were identified for 1H_NIL1_S, 1H_NIL2_S and 1H_NIL3_S, respectively. Due to limited number of DEGs identified, no common enriched GO terms across pairwise comparisons for genes down-regulated during infection in ‘R’ or ‘S’ isolines were detected. GO term enrichment lists were compared to find terms commonly enriched across all three ‘R’ or ‘S’ isolines. For genes up-regulated during infection, three GO terms relating to the Cytochrome P450 superfamily (iron ion binding (GO:0005506), heme binding (GO:0020037) and tetrapyrrole binding (GO:0046906) were overrepresented consistently in both ‘R’ and ‘S’ isolines. In addition, glutathione transferase activity (GO: 0004364) was enriched across all three ‘S’ isolines and enriched across two ‘R’ isolines. GO terms enriched in only the three ‘R’ isolines or ‘S’ isolines were not detected. Results from the enrichment analysis inferred a common response to infection in both ‘R’ and ‘S’ isolines with terms having known roles in both biotic and abiotic stress responses. However, specific processes showing a consistent difference between ‘R’ and ‘S’ isolines which might explain increased resistance in ‘R’ isolines were not found at this relatively early infection timepoint.

**Fig. 3:**
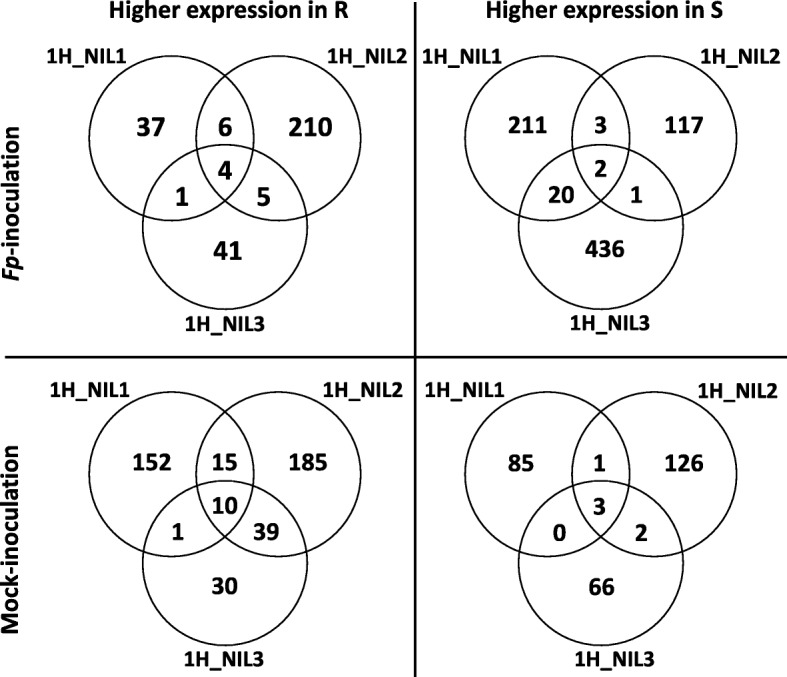
DEGs between ‘R’ and ‘S’ isolines under *Fp-* (R^I^_vs_S^I^) or mock-inoculation (R^M^_vs_S^M^). Venn diagrams show the numbers of DEGs up-regulated in ‘R’ (left) or ‘S’ (right) isolines under *Fp-* (up) or mock- inoculation (down). DEGs were determined with the threshold of FDR ≤ 0.05 and |log_2_ fold-change| ≥ 1 or ‘inf’ (one of the comparative objects did not express and the other did)

To assess transcriptomic responses to FCR infection mediated by *Qcrs.cpi-1H*, we compared DEGs between the ‘R’ and ‘S’ isolines. These comparisons found that a total of 303 genes were up-regulated and 790 down-regulated from the *Fp*-inoculation treatment (Table 2). Only 4 of the up-regulated genes and 2 of the down-regulated ones were shared by all three NIL pairs (Figs. 1 and 3). Of the DEGs identified from the mock-inoculated samples, 440 were up-regulated and 283 down-regulated (Table 2). Ten of the up-regulated and 3 down-regulated ones were shared across all the three comparisons (Fig. 3).

### SNPs between the ‘R’ and ‘S’ isolines across the three 1H_NIL pairs

**Fig. 4:**
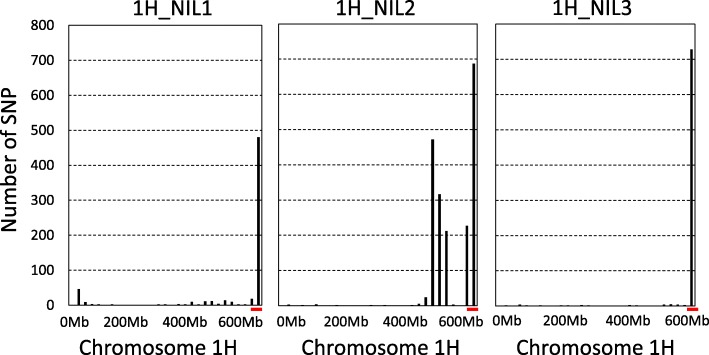
Distribution of SNPs in the expressed genes along chromosome 1H in three pairs of the 1H_NILs. Vertical axis shows number of SNPs. Horizontal axis shows chromosome 1H from short (left) to long (right) arm in base pairs (bp). Red bars represent the candidate region harbouring the FCR resistant locus *Qcrs.cpi-1H*

**Fig. 5:**
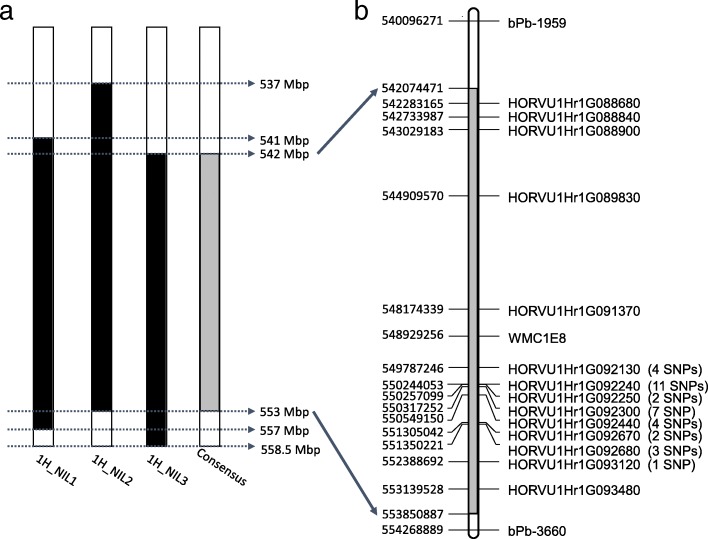
Physical distribution of DEGs within the consensus SNP-enriched region. **a** The physical range of SNP-enriched regions. Black boxes indicate the regions defined by SNPs within each 1H_NIL pair; the grey box represents the consensus region. **b** Physical distribution of DEGs shared among the three comparisons within the consensus region. The initial QTL region was flanked by *bPb-1595* and *bPb-3660*. SNP-up/down indicate the borders of the consensus region. The numbers of SNPs identified within genes were in brackets

In total, 2753 non-redundant homozygous SNPs were detected between the ‘R’ and ‘S’ isolines. The number of SNPs detected from 1H_NIL2 was more than twice compared with those detected from either of the other two NIL pairs. Of these SNPs, 293 were common among the three pairs of the 1H_NILs. As expected, the majority of the SNPs shared among the three NIL pairs located at the distal end of chromosome arm 1HL where *Qcrs.cpi-1H* resides (Fig. 4). They spanned a physical distance of ~ 11.0 Mbp (Fig. 5a).

### DEGs with SNPs between the resistant and susceptible isolines targeting the *Qcrs.cpi-1H* locus

Based on the reference genome of barley cv. Morex, 266 HC genes were identified within the common interval across three 1H_NIL pairs. Among these HC genes, 56 contained SNPs and 14 were differentially expressed between the isolines for at least one of the NIL pairs (Fig. 5b; Additional file 3: Table S3). Notably, five protein-coding genes were not only differentially expressed across the three NIL pairs but also carried SNPs led to non-synonymous variations (Table 3 and additional file 4: Table S4). These protein-coding genes should form the primary targets in identifying candidate genes underlying FCR resistance at this locus.

**Table 3:** Expression patterns of five DEGs bearing non-synonymous SNPs located in the interval harbouring the FCR resistant locus *Qcrs.cpi-1H*

Gene ID	Gene Description ^a^	Number of Non-synonymous SNPs	Pattern of differential expression
HORVU1Hr1G092130	WRKYDNA-binding protein 23	1	Upregulated in 3 S isolines post inoculation
HORVU1Hr1G092240	Glucan endo-1,3-beta-glucosidase13	4	Upregulated in 3 R isolines post inoculation
HORVU1Hr1G092250	Receptor-like kinase	1	Upregulated in 3 R and 3 S isolines post inoculation
HORVU1Hr1G092300	Receptor-like kinase	6	Upregulated in 3 R post inoculation
HORVU1Hr1G092440	P-loop containing nucleoside triphosphate hydrolases super family protein	4	Upregulated in 3 S isolines post inoculation

^a^ Gene descriptions were retrieved from the annotation file of the genome of barley cv. Morex

## Discussion

FCR is a chronic disease for cereal production in semi-arid regions worldwide. It has long been recognised that breeding and growing resistant varieties have to form an integral part in the effect of effectively reducing damages from the disease. Previous studies also show that strong interactions between FCR severity and several characteristics including flowering time and plant height exist thus QTL detected from mapping populations need to be validated. In the study reported here, we successfully validated the QTL on chromosome arm 1HL by developing and assessing NILs targeting the locus. DEGs with SNPs shared by three pairs of the NILs further delineated the locus to an interval of about 11.0 Mbp. They would be invaluable for fine mapping the locus and cloning the gene(s) underlying its resistance. SNPs in several of the DEGs lead to amino acid changes and they would be primary targets in investigating the mechanism of FCR resistance.

It is of note that significant variation was found in the numbers of DEGs detected among the three pairs of NILs assessed. Previous studies showed that FCR development can be affected by various characteristics including plant height [11, 17, 21, 31] and flowering time [12, 16, 32]. Each of the NIL pairs used in this study was developed from a different heterozygous plant based on the profile of a single marker. This method ensured that different NIL pairs, including those from the same population, would have different genetic backgrounds. The different genetic backgrounds would lead to difference in FCR development at any given time point. In other words, although symptom of FCR infection was not visually observable for any of the NILs at 4 dpi when the samples for RNA-seq were taken, the advancement of FCR development among them must be different.

The interactions between FCR severity and other characteristics may also contributed to the difference in the effects of the 1HL locus between the use of NILs as described in this study and that based on QTL mapping [10]. In addition to the targeted trait, many other characteristics likely also segregate in populations routinely used for QTL mapping. They include populations of recombinant inbred lines and doubled haploid lines. In essence, a targeted locus is always assessed in different genetic backgrounds in QTL mapping studies, making its accurate assessment difficult. In the contrary, the two isolines forming each NIL pair differ mainly by the targeted locus. The fact that assessments for any characteristics can be carried out by comparing two isolines only must also contribute to the likelihood that more accurate assessment can be achieved by using NILs.

It is also of note that significant difference in FCR resistance was not detected between isolines for three of the eight pairs of putative NILs developed in this study. Different from the method of using markers flanking the targeted locus [33], we used only one linked marker obtained from a QTL mapping study [10] in developing the NILs. As discussed in earlier reports [22, 23], the approach of using a single linked marker is preferred as it should reduce the sizes of chromosomal segments differentiating the isolines for NILs obtained. However, QTL mapping studies have only limited resolution [34] thus markers obtained from such studies may not be tightly linked with a targeted locus. Clearly, recombination between the linked marker and its target may occur, resulting in false NILs.

Within the targeted interval of the 1HL locus, five protein-coding genes are highly interesting due not only to their patterns of expression among the NILs but also the fact that they contain nonsynonymous SNPs. They are known to be involved in plant-pathogen interaction or abiotic stress (i.e. drought) which facilitates *F. pseudograminearum* infection. They include the two receptor-like kinase (RLK) genes (*HORVU1Hr1G092250* and *HORVU1Hr1G092300*) which are involved in the immune systems in various plant species [35]. RLK locates on either the plasma or cytoplasmic membrane and are responsible for recognizing elicitor, usually small secreted protein, generated by pathogens. The perception of elicitor often triggers a fierce hypersensitive response which can cause programmed cell death [36]. Another one is the gene for glucan endo-1,3,-beta-glucosidase (*HORVU1Hr1G092240*) which plays an important role in defence against pathogen infection [37]. Its expression has been detected in the response to biotic stress in various plant species [38, 39]. *HORVU1Hr1G092440* encoding a P-loop containing nucleoside triphosphate hydrolases (P-loop NTPase) protein is also among the DEGs with SNPs located in the targeted interval. Previous results showed that this gene negatively regulates plant defence response in both rice and *Arabidopsis* [40, 41]. Once bonded with ATP, *OsYchF1*, a P-loop NTPase in rice, contributes to resistance to biotic stress [42].

It is also interesting to note that one of the DEGs with SNPs located in the targeted interval confers tolerance to drought. This is *HORVU1Hr1G092130* which codes a WRKY transcription factor which plays a key role in signalling in the defense response to biotic and abiotic stress [43, 44]. A homolog of *HORVU1Hr1G092130* in rice, *Os05g0583000* was strongly induced during drought response [45]. Over-expression of *Os05g0583000* coding sequence in *Arabidopsis* provided improved drought tolerance [46]. The presence of this gene related to drought tolerance is not a surprise as the relationship between drought stress and *Fusarium* crown rot severity in agricultural systems has been well documented. FCR causes severe yield loss mainly in semi-arid regions [1] and drought stress forms part of the procedures in FCR assay, which was also performed in the current study, in both wheat [47, 48] and barley [10, 12, 13]. As such, it is not unexpected that the causal gene of *Qcrs.cpi-1H* may decrease FCR disease expression through improved drought stress tolerance rather than classical disease resistance mechanisms.

Based on the DEGs detected in this work, it also seems unlikely that the mechanism for resistance is driven by differences in classical resistance mechanisms previously described as important for defence against *Fusarium* pathogens (Additional file 5: Table S5). The *Fusarium* mycotoxin, deoxynivalenol has been shown to be required for full virulence of *F. pseudograminearum* when infecting wheat and *Brachypodium* [49, 50]. Detoxification of deoxynivalenol has been strongly implicated in defence against *F. graminearum* causing *Fusarium* head blight with DON detoxifying UDP glycosyltransferases identified in wheat, barley and *Brachypodium* [51–53]. The UDP-glycosyltransferase detoxifying DON in barley (*HORVU5Hr1G047150*) [52, 54] was not found to be differentially expressed between or showing SNPs differences between R or S isolines in the current study (Additional file 5: Table S5). Previous studies have also shown that induced systemic resistance mechanisms are involved in response to *F. pseudograminearum* infection [55]. Key markers for systemic acquired or induced systemic resistance, such as genes encoding jasmonate biosynthetic enzymes, salicylic acid biosynthetic enzymes and pathogenesis related proteins, were differentially expressed in response to infection across both resistant and susceptible isolines to similar magnitudes (Additional file 5: Table S5). Therefore, from comparison of molecular responses observed in resistant and susceptible isolines, we did not find any inference that the effect of the 1HL locus occurs through previously characterised quantitative resistance mechanisms. We thus conclude that resistance mediated by the 1HL resistance locus may provide a highly novel FCR resistance source in barley.

## Conclusions

In this study*,* we developed five pairs of NILs targeting the FCR resistance locus *Qcrs.cpi-1H*. Phenotyping these NIL found that the resistant allele at *Qcrs.cpi-1H* could significantly reduce FCR severity. Gene expression and SNP analysis of transcriptomic data derived from three pairs of the 1H_NILs delineated the *Qcrs.cpi-1H* locus into an about 11 Mbp interval containing 56 genes with SNP(s). Of these genes, five DEGs bearing non-synonymous SNPs form primary targets in identifying gene(s) underlying the *Qcrs.cpi-1H* locus.

## Materials and methods

### Development of near isogenic lines

The heterogeneous inbred family method [56], combined with the fast-generation technique [57], was used to develop NILs targeting the 1HL locus (*Qcrs.cpi-1H*). Plants were raised in glasshouses at Queensland Bioscience Precinct in Brisbane, Australia. Heterozygous plants were identified from two segregating populations, ‘Locker//AWCS079/AWCS276’ and ‘Commander//AWCS079/AWCS276’, using the SSR marker *WMC1E8.* This marker was one of those linked closely with *Qcrs.cpi-1H* identified from QTL mapping [10]. Primer sequences of the marker were: forward 5′-TCATTCGTTGCAGATACACCAC-3′; and reverse 5′-TCAATGCCCTTGTTTCTGACCT-3′. The identified plants were self-pollinated for eight generations and a single pair of putative NILs was then selected from each of the original heterozygous plants.

### FCR inoculation and assessment

FCR inoculation was conducted in the controlled environment facilities (CEFs) at Queensland Bioscience Precinct, Brisbane. Four independent trials were conducted against the putative NILs. Each trial consists of two replicates and 14 seedlings per isoline were used in each of the replicates. A highly aggressive isolate of *F. pseudograminearum* (CS3096) was used for inoculation in these trials. This isolate was collected in northern New South Wales and maintained in the CSIRO collection [58]. Procedures used for inoculum preparation, inoculation and FCR assessment were based on those described by Li et al. [59]. Briefly, seeds were surface-sterilized by treating with 2% hypochlorite solution for 10 min and then thoroughly rinsed with distilled water for four times. The seeds were then germinated on three layers of filter paper saturated with water in petri-dishes. Newly germinated seedlings (with coleoptile lengths ranging from 0.5 to 1.0 cm) were inoculated by immersing in *Fusarium* spore suspension (or water for controls) for 1 min. Two treated seedlings were sown in a 4 cm × 4 cm square punnet (Rite Grow Kwit Pots, Garden City Plastics, Australia) containing autoclaved potting mix. Fifty-six punnets were placed in a plastic seedling tray for easy handling. Inoculated seedlings were kept in CEFs. Settings for the CEFs were: 25/16(± 1) °C day/night temperature and 65%/85% day/night relative humidity, and a 14-h photoperiod with 500 mol m − 2 s − 1 photon flux density at the level of the plant canopy. Plants were watered only when wilt symptoms appeared. FCR severity for each plant was assessed with a 0–5 scale, where “0” standing for no symptom and “5” representing whole plant necrotic [59]. Disease indices (DI) was calculated for each line following the formula of DI = (∑_n_X / 5 N) × 100, of which, *X* is the scale value of each plant, *n* is the number of plants in the category, and *N* is the total number of plants assessed for each line. The difference between the isolines possessing the resistant and susceptible allele for each of the putative NIL pairs was assessed with the student *t* test.

### RNA extraction and sequencing

Samples for RNA sequencing were obtained from three pairs of the NILs. Inoculation was conducted with either the *F. pseudograminearum* isolate (*Fp*-inoculation) or distilled water (mock) following the protocol described above. Three biological replications were conducted for every isolines. Each replication consisted of seven seedlings. Tissues for RNA extraction were collected by cutting the shoot bases (2 cm) at 4 days post inoculation (dpi) and snap-frozen in liquid nitrogen and kept at − 80 °C until processed. The time point for sampling was selected based on a previous study [29].

A total of 36 samples were obtained from the six isolines. Samples were crushed into fine powder and RNA extraction was conducted using an RNeasy plant mini kit (Qiagen, Hilden, Germany) according to manufacturer’s instructions (including DNase-I digestion). The yield and purity of RNA samples were measured using a Nanodrop-1000 Spectrophotometer. The integrity of all RNA samples was assessed by running the total RNA on 1% agarose gels. RNA sequencing was carried out by the Australian Genome Research Facility Ltd. (Parkville, Victoria, Australia) and 100-bp paired-end reads were produced using the Illumina Hiseq-2000. All 36 RNA-seq libraries were run across four lanes of a HiSeq2000.

### Transcriptomic analyses

Commands used for trimming raw data and analysing trimmed reads were described by Habib et al. [29]. FastQC (version 0.11.2) was used as a preliminary check for PHRED scores. Raw reads were trimmed using the SolexaQA package (version 3.1.3) with a minimum PHRED quality value of 30 and minimum length of 70 bp. TopHat2 (version 2.0.13) [60] was used to map filtered reads to the barley cv. Morex genome (https://webblast.ipk-gatersleben.de/barley_ibsc/downloads/:150831_barley_pseudomolecules) which is now widely used as the reference for barley [61].

#### Differential gene expression analysis

Cufflinks (version 2.0.2) [60] was used to assemble the mapped reads. DEGs were identified with Cuffdiff from the Cufflinks tool package with high-confidence genes annotated in the ‘Morex’ genome. Fragments per kilobase of exon per million mapped reads (FPKM) was applied for each transcript to represent the normalized expression value. The fold change in gene expression was calculated according to the equation: Fold Change = log_2_ (*FPKM*_*A*_/ *FPKM*_*B*_).

Pairwise comparisons were conducted between different treatments for the same isoline (S^M^_v_S^I^ and R^M^_v_R^I^) and between isolines under *Fp-*inoculation (S^I^_v_R^I^) or mock-inoculation (S^M^_v_R^M^) (Fig. 1). ‘M’ stands for ‘mock-inoculation’, ‘I’ for *Fp*-inoculation, ‘S’ for susceptible isolines, and ‘R’ resistant isolines. DEGs were determined with the adjusted *p*-value threshold of ≤0.05 and log_2_ fold change of ≥1 or ≤ − 1 or ‘inf’ (where the FPKM value in one dataset is zero and the other is not). Venny 2.0 was used for Venn diagram analysis [62].

#### Validation of differentially expressed genes using qRT-PCR

Three genes (*HORVU1Hr1G092240*, *HORVU1Hr1G092250* and *HORVU1Hr1G092300*; primer sequences were listed in Additional file 1: Table S1) were selected from the identified DEGs for validation. Quantitative real-time PCR (qRT-PCR) was used for validation with the actin protein gene as the internal housekeeping reference (forward primer: 5′-GCCGTGCTTTCCCTCTATG-3′; reverse primer 5′-GCTTCTCCTTGATGTCCCTTA-3′). Inoculation, tissue sampling and RNA extraction were carried out using the aforementioned methods. Three biological replicates, each with two technical replications, were used for each genotype-treatment sample per isoline.

The procedures for synthesising cDNA and qRT-PCR were conducted following the methods described by Ma et al. (2013). The relative fold changes were calculated using the comparative CT method (2^-∆∆CT^). The average value of the two technical replications was used to represent the biological replicate for each of the samples.

#### SNP calling and nonsynonymous variation identification

For each genotype, all six sequence files (three biological replicates by two treatments) were concatenated after removing low-quality sequences. The concatenated files were then aligned to the ‘Morex’ genome using Biokanga align with a maximum of two mismatches per read. SNPs between the ‘R’ and ‘S’ isolines of each NIL pair were identified using the Biokanga snpmarkers with a minimum 80% score (the percentage of a given nucleotide at an SNP position is at least 80% in the ‘R’ or ‘S’ isoline). The SNPs were annotated using snpEff 4.3q and the variant database was built based on the Morex genome and its annotation file [61].

### Gene annotation and GO term enrichment analysis

BLAST, mapping and annotation steps were performed using the standard parameters in BLAST2GO [63] and the GO annotation results were used as reference (Additional file 6: Table S6) in the following analysis. DEGs identified from all comparisons were separated into up-regulated and down-regulated gene lists (Additional file 6: Table S6) and submitted to singular enrichment analysis using agriGO [64, 65] with default setting.

## Additional files

Additional file 1: **Table S1.** Primer sequences and results of qPCR validation of RNA-Seq experiments. qPCR results for 3 selected DEGs between the mock and inoculated isolines among the three pairs of NILs. The fold-change of qPCR results for each gene was generally in agreement with RNA-seq results. (XLSX 10 kb)

Additional file 2: **Table S2.** Enriched GO terms associated with DEGs and HEGs. In the comparison column, ‘M’ = mock-inoculation; ‘I’ = *Fp*-inoculation; ‘R’ = resistant isoline; ‘S’ = susceptible isoline. ‘O’ column stands for three domains, ‘C’ = cellular component; ‘F’ = molecular function; ‘P’ = biological process. ‘#list’ means the number of term-specific genes from the input list. ‘#bg’ means the number of term-specific genes from the background. FDR < 0.05. (XLSX 18 kb)

Additional file 3: **Table S3.** DEGs and SNP-bearing genes within the SNP consensus region across the three NIL pairs. Log2Fold Changes for each of the genes in different comparisons were listed (FDR < = 0.05). ‘M’ = mock-inoculation; ‘I’ = *Fp*-inoculation; ‘R’ = resistant isoline; ‘S’ = susceptible isoline. Positive values mean that the gene was up-regulated following *Fp*-inoculation; and negative values indicate down-regulated genes. ‘inf’ means the value of the comparative object is zero. (XLSX 15 kb)

Additional file 4: **Table S4.** Annotation of non-synonymous SNPs in genes within the consensus region. $: “-” means that SNPs were not found in the high confidence (HC) gene. * blank cell means no amino acid change was detected. (XLSX 20 kb)

Additional file 5: **Table S5.** DEGs related to typical resistance mechanisms against *F. graminearum and F. pseudograminearum*. Log2Fold Changes for each gene in different comparisons were listed (FDR < = 0.05). ‘M’ = mock-inoculation; ‘I’ = *Fp*-inoculation; ‘R’ = resistant isoline; ‘S’ = susceptible isoline. Positive values mean that the gene was up-regulated following *Fp*-inoculation; negative value indicates down-regulated genes, and ‘inf’ means the value of the comparative object is zero. (XLSX 15 kb)

Additional file 6: **Table S6.** GO annotations of up- (Sheet 1) and down-regulated (Sheet 2) DEGs and background references (Sheet 3) used in GO enrichment analysis. (XLSX 9087 kb)

## Data Availability

RNA sequences used in this study were available at the National Centre for Biotechnology Information (NCBI) with the accession number of PRJNA541021. The other supporting data were included as additional files.

## Abbreviations

CEF: Controlled environment facilities
DEG: Differentially expressed gene
DI: Disease index
FCR: *Fusarium* crown rot
*Fp*: *Fusarium pseudograminearum*
FPKM: Fragments Per Kilobase of exon per Million mapped reads
GO: Gene ontology
HC: High confidence
NIL: Near-isogenic line
P-loop NTPase: P-loop containing nucleoside triphosphate hydrolases
RLK: Receptor-like kinase
SNP: Single nucleotide polymorphism
